# Stanniocalcin 2 Regulates Non-capacitative Ca^2+^ Entry and Aggregation in Mouse Platelets

**DOI:** 10.3389/fphys.2018.00266

**Published:** 2018-03-23

**Authors:** Esther López, L. Gómez-Gordo, Carlos Cantonero, Nuria Bermejo, Jorge Pérez-Gómez, María P. Granados, Gines M. Salido, Juan A. Rosado Dionisio, Pedro C. Redondo Liberal

**Affiliations:** ^1^Department of Physiology (PHYCELL) of the Veterinary Faculty, University of Extremadura, Cáceres, Spain; ^2^Department of Animal Medicine, Veterinary Faculty University of Extremadura, Cáceres, Spain; ^3^Hematology Unit, San Pedro de Alcantara Hospital, Cáceres, Spain; ^4^Faculty of Sport Sciences, University of Extremadura, Cáceres, Spain; ^5^Aldea Moret Health Center, Extremadura Health Service, Cáceres, Spain; ^6^Institute of Molecular Pathology Biomarkers, Cáceres, Spain

**Keywords:** STC2, platelets, non-SOCE, Orai3, TRPC6, TRPC3

## Abstract

Stanniocalcin 2 (STC2) is a fish protein that controls body Ca^2+^ and phosphate metabolism. STC2 has also been described in mammals, and as platelet function highly depends on both extracellular and intracellular Ca^2+^, we have explored its expression and function in these cells. STC2^−/−^ mice exhibit shorter tail bleeding time than WT mice. Platelets from STC2-deficient mice showed enhanced aggregation, as well as enhanced Ca^2+^ mobilization in response to the physiological agonist thrombin (Thr) and the diacylglycerol analog, OAG, a selective activator of the non-capacitative Ca^2+^ entry channels. Interestingly, platelets from STC2^−/−^ mice exhibit attenuated interaction between STIM1 and Orai1 in response to Thr, thus suggesting that STC2 is required for Thr-evoked STIM1-Orai1 interaction and the subsequent store-operated Ca^2+^ entry (SOCE). We have further assessed possible changes in the expression of the most relevant channels involved in non-capacitative Ca^2+^ entry in platelets. Then, protein expression of Orai3, TRPC3 and TRPC6 were evaluated by Western blotting, and the results revealed that while the expression of Orai3 was enhanced in the STC2-deficient mice, others like TRPC3 and TRPC6 remains almost unaltered. Summarizing, our results provide for the first time evidence for a role of STC2 in platelet physiology through the regulation of agonist-induced Ca^2+^ entry, which might be mediated by the regulation of Orai3 channel expression.

## Introduction

Platelets are anucleated cells that play a relevant role in haemostasis. Attenuation of platelet function causes spontaneous browsing and bleeding. In contrast, hyperaggregability might lead to both macrothrombosis (observed in ictus or stroke) and microthrombosis (as detected in certain forms of retinopathy and nephropathy), as well as other cardiovascular disorders (Gue and Gorog, 2017; Malerba et al., 2017; Mancuso and Santagostino, 2017; Scherlinger et al., 2017). Hence, understanding the mechanisms underlying platelet function would be of relevance to prevent platelet-related complications linked to certain diseases. It is well known that platelet function strongly depends on changes in the intracellular calcium concentration ([Ca^2+^]_c_). Agonist might increase [Ca^2+^]_c_ either by releasing Ca^2+^ from the intracellular stores or inducing Ca^2+^ entry from the extracellular medium. Regarding Ca^2+^ entry, store-operated calcium entry (SOCE) is a major mechanism activated by receptor occupation (Redondo and Rosado, 2015; Berna-Erro et al., 2016). With respect to SOCE activation, it is well known that a reduction in the endoplasmic reticulum Ca^2+^ content is detected by STIM1, which undergoes a conformational change and translocation to endoplasmic reticulum/plasma membrane (ER/PM) junctions in order to communicate with and activates Orai1 (Schindl et al., 2009; Scrimgeour et al., 2014; Shim et al., 2015). Since new regulators of the core components conforming the structure of SOCE channels has been described, more research on this topic is required for fully understanding the mechanism that controls Ca^2+^ entry from the extracellular medium. Orai1 is the pore-forming subunit of the highly Ca^2+^ selective calcium release-activated calcium (CRAC) channels (Prakriya et al., 2006). Additionally, STIM1 also activates a non-selective current mediated by store-operated calcium (SOC) channels, which consist on Orai1 and TRPC1 subunits (Desai et al., 2015). SOCE has been found to be modulated by a number of regulatory proteins that fine-tune Ca^2+^ influx in order to generate appropriate Ca^2+^ signals, and can be regulated by direct protein interaction (like the cytosolic Ca^2+^ sensor, calmodulin; Galan et al., 2011; Li et al., 2017).

In addition to SOCE, non-capacitative Ca^2+^ entry has also been reported in platelets, where TRPC3, TRPC6 and Orai3 have been found to play an important role (Berna-Erro et al., 2012; Harper et al., 2013). Concerning Orai3, this channel is able to form store independent channels with Orai1 regulated by arachidonate, the arachidonic acid-regulated calcium-selective (Mignen et al., 2008; Demuro et al., 2011; Zhang et al., 2014; Albarran et al., 2016) as well as the supposedly homomeric channels regulated by 2-aminoethoxydiphenyl borate (Demuro et al., 2011; Amcheslavsky et al., 2014). TRPC6 has been found to be involved in both SOCE and non-capacitive Ca^2+^ entry in human platelets and TRPC3 was reported to participate in non-capacitative Ca^2+^ entry in human and murine platelets (Zbidi et al., 2009; Harper et al., 2013).

Stanniocalcin 2 (STC2) was first identified as a fish protein involved in calcium and phosphate homeostasis (Wagner et al., 1998a,b). In mammals, indirect evidence suggest that STC2 has a relevant regulatory role on phosphate physiology. Contrary, its regulatory function in Ca^2+^ is limited to the intracellular environments, as evidenced in bone tissue (Ishibashi and Imai, 2002). In addition, STC2 is overexpressed by cancer cells (Na et al., 2015; Wang et al., 2015). Mammalian STC2 has been reported to play a protective role against oxidative stress or hypoxia (Law and Wong, 2010; Kim et al., 2015). Both stressing conditions evoke the accumulation of unfolded proteins in the ER leading to the activation of the unfolded protein response (UPR) to which STC2 belongs (Ito et al., 2004). Ca^2+^ mobilizing agents, such as thapsigargin (TG) and Ca^2+^ ionophores, have been found to activate UPR in culture cells (Ito et al., 2004), thus promoting the transcription of a variety of genes, including *NUCB2, ATP2A2, CGRP2*, and *STC2* (Ito et al., 2004). In mouse embryonic fibroblasts STC2 overexpression results in reduced SOCE probably mediated by interaction with STIM1 (Zeiger et al., 2011), which strongly suggests a possible role for STC2 in the regulation of SOCE. We have recently reported that STC2 is expressed in human and mouse platelets (Lopez et al., 2017). Here we provide for the first time evidence for a role of STC2 in the regulation of Ca^2+^ entry and aggregation in mouse platelets. Our results indicate that in STC2-deficient mice thrombin-stimulated platelet aggregation and Ca^2+^ mobilization is enhanced, which might be associated to the overexpression of Orai3 channels.

## Materials and methods

### Materials

Fura-2/AM was from Invitrogen Molecular probes (California, USA). Mouse anti-STIM1 [Clone 44 Clone 44–GOK (RUO)] antibody was from BD Biosciences® (Madrid, Spain). Rabbit polyclonal anti-TRPC1 (against peptide QLYDKGYTSKEQKDC) antibody was supplied by OriGene® (Rockville (MD) USA). Anti-TRPC6 extracellular antibody was from Alomone (Jerusalem, Israel). Anti-TRPC3 was from Abcam (Cambridge, UK). Mouse and rabbit HRP-conjugated secondary antibodies were from Jackson Immunoresearch® (West Grove, PA, USA). Rabbit polyclonal anti-Orai1 (against sequence amino acids 288–301) antibody, anti-Orai3 antibody, anti-actin antibody, thrombin (Thr), ADP, thapsigargin (TG), 1-oleoyl-2-acetyl-sn-glycerol (OAG), apyrase, aspirin, as well as other reagents of analytic grade were purchased from Sigma-Aldrich® (Madrid, Spain).

### Animals

C57BL/6J WT mice were provided by Harlam®, while C57BL/6J STC2^−/−^ mice were kindly provided by Dr. Roger Reddel from the Children's Medical Research Institute (CMRI) (Australia) (Chang et al., 2008). Animals were breed under controlled environmental conditions (25°C), which consisted on a regular 12 h light-dark photoperiod and free access to drink and food. The two mice conolies were bred separately, and after two or three generations, animals were backcrossed in order to minimize genetic drift. STC2 deficiency was regularly assessed by PCR and Western blotting. Mice of age range between 8 and 10 weeks were bled three times under isoflurane anesthesia, leaving a resting period of a week between each extraction.

All procedures were performed in agreement with the Helsinki Declaration, the guidelines from the Council of Europe N° 123 (Strasbourg, 1985) and have been approved by the Local Ethical Committee.

### Tail bleeding

Anesthetized animals were subjected to surgical sectioning of the tip of the tail (sections of 1–4 mm were cut off, approximately), and a whatman filter paper was imbibed with the blood at regularly periods of 30 s until the bleeding stopped (Vermeersch et al., 2017). In case of excessive bleeding, lasting more than 25 min, the experiments were artificially interrupted by cauterizing the injury. Average of the bleeding time was considered in these experiments, and the possible differences between WT and STC2-deficient mice were analyzed.

### Blood extraction procedures and platelet isolation

Isoflurane anesthetized mice were bled from the retroorbital plexus (Berna-Erro et al., 2014), and the blood was mixed with 300 μL of acid citrate dextrose buffer containing: 85 mM sodium citrate, 78 mM citric acid, 111 mM glucose, pH 7.3. Blood was centrifuged at 300 × *g* for 5 min, and upon collected the supernatants, they were centrifuged again at 100 × *g* for 5 min in order to obtain the platelet-rich plasma (PRP). PRP was supplemented with aspirin (100 μM) and apyrase (40 U/mL) (except those used for aggregation, to which only apyrase was added), and PRP was centrifuged again at 600 × *g* for 5 min. The pellet was suspended in Ca^2+^-free Tyrode's buffer containing: NaCl 137 mM, KCl 2.7 mM, NaHCO_3_ 12 mM, NaH_2_PO_4_ 0.43 mM, Glucose 0.1%, HEPES 5 mM, BSA 0.35%, MgCl_2_ 1 mM, pH 7.13 and supplemented with apyrase (40 μg/mL).

### Platelet aggregation

Platelet aggregation was monitored using a Cronologh® aggregometer according to the protocols described elsewhere (Lopez et al., 2013a). Briefly, isolated platelets were suspended in fresh-Tyrode's buffer containing 40 U/mL of apyrase. At the moment of the experiment 1 mM CaCl_2_ was added and following aggregation was stimulated by using physiological agonists such as thrombin (Thr, 0.1 U/mL) and ADP (100 μM). Percentage of aggregation at two different time points (3 and 8 min after stimulation with Thr, and 4 and 10 min after treatment with ADP) as well as the percentage of the slope (in case of Thr) and percentage of delay time to initiate the irreversible phase of aggregation (in case of ADP) were used to compare platelet aggregability in both mice strains.

### [Ca^2+^]_c_ determination

Platelets suspended in Tyrode's buffer were loaded with fura-2 by incubating for 30 min at room temperature with fura-2/AM (2 μM) (Lopez et al., 2013b; Berna-Erro et al., 2014). Fluorescence was recorded from aliquots of magnetically stirred platelet suspensions at 37°C using a fluorescence spectrophotometer (RF-5301PC, Shimadzu Corporation, Kyoto, Japan) with excitation wavelengths of 340 and 380 nm and emission at 505 nm. Changes in [Ca^2+^]_c_ were monitored using the fura-2 340/380 fluorescence ratio and calibrated according to the method of Grynkiewicz and coworkers (Grynkiewicz et al., 1985). Thr-, OAG- and TG-evoked Ca^2+^ mobilization was estimated as the integral of the rise in [Ca^2+^]_c_ above basal for 2½ min after the addition of the stimuli, taking a sample every second and expressed as nM•s (Lopez et al., 2005). To calculate the initial rate of Ca^2+^ elevation after the addition of TG to the medium, the traces were fitted to the equation y = A + *K*X, where *K* is the slope (Redondo et al., 2003).

### Immunoprecipitation and western blotting

Murine platelets were either mixed with RIPA (pH 7.2, containing 474 mM NaCl, 30 mM Tris, 3 mM EGTA, 0.3% SDS, 3% sodium deoxycholate, 3% triton X-100, 3 mM Na_3_VO_4_ and protease inhibitors) or Laemmli's buffer as required (Alcaraz et al., 1990; Karlsson et al., 1994). Platelet samples in RIPA were immunoprecipitated by incubation with 2 μg/mL of anti-Orai1 and anti-STIM1, and protein A-agarose beads overnight at 4°C. The immunoprecipitates were resolved by 10% SDS/PAGE, and separated proteins were electrophoretically transferred onto nitrocellulose membranes using a semi-dry blotter (for 2 h at 0.8 mA/cm^2^; Hoefer Scientific, Newcastle under-Lyme, U.K.). Blots were incubated overnight with 10% (w/v) BSA in Tris-buffered saline with 0.1% Tween 20 (TBST) to block residual protein-binding sites. Detection of STIM1, Orai1, TRPC1, TRPC3, TRPC6, and Orai3 was achieved by incubation for 1 h with anti-STIM1 antibody diluted 1:1,000 in TBST, for 2 h with anti-Orai1, anti-TRPC1, or anti-TRPC6 antibody diluted 1:500 in TBST, and overnight with anti-TRPC3 or anti-Orai3 antibody diluted 1:1,000 and 1:250 in TBST, respectively. To detect the primary antibody, membranes were incubated with the appropriate secondary antibody (diluted either 1:5,000 or 1:10,000 in TBST for 1 h) washed six times in TBST, and exposed to enhanced chemiluminescence reagents for 1 min. Optical density of the bands was detected using the C-Digit System (Licor®, Spain), and differences between samples were analyzed using the Image J software (N.I.H. USA). Data are normalized using the amount of actin (for Western blotting) or the result obtained after reprobing the membranes with the immunoprecipitating antibody (for the immunoprecipitations).

### Statistical analysis

Analysis of statistical significance was performed using the Student's unpaired *t*-test or one-way analysis of variance (ANOVA) combined with the Dunnet's post-test. The significance level was *P* < 0.05.

## Results

### STC2-deficient mice exhibit shorter bleeding times

**Figure 1:**
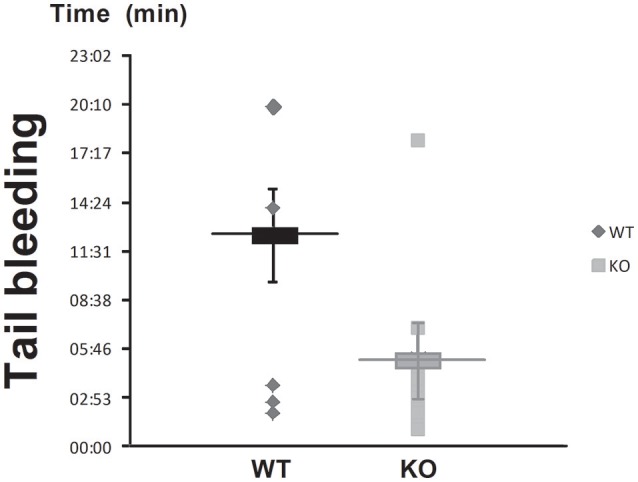
STC2 is involved in blood clotting. The tip of the tail of anesthetized STC2-deficient (KO) and WT mice was sectioned as described in Materials and Methods, and the bleeding time was determined. Box graph represents mean ± SEM of 10 to 14 mice.

Wild type (WT) and STC2-deficient mice (KO) were subjected to tail bleeding as described in the Material and Methods section. As depicted in Figure 1, STC2^−/−^ mice presented shorter mean bleeding time than WT mice (5:10 ± 1:31 vs. 12:28 ± 2:40 min:s, respectively; *n* = 10–14, *P* < 0,001). These findings suggest that STC2 might play a role in the regulation of haemostasia.

### STC2 is involved in murine platelet aggregation

**Figure 2:**
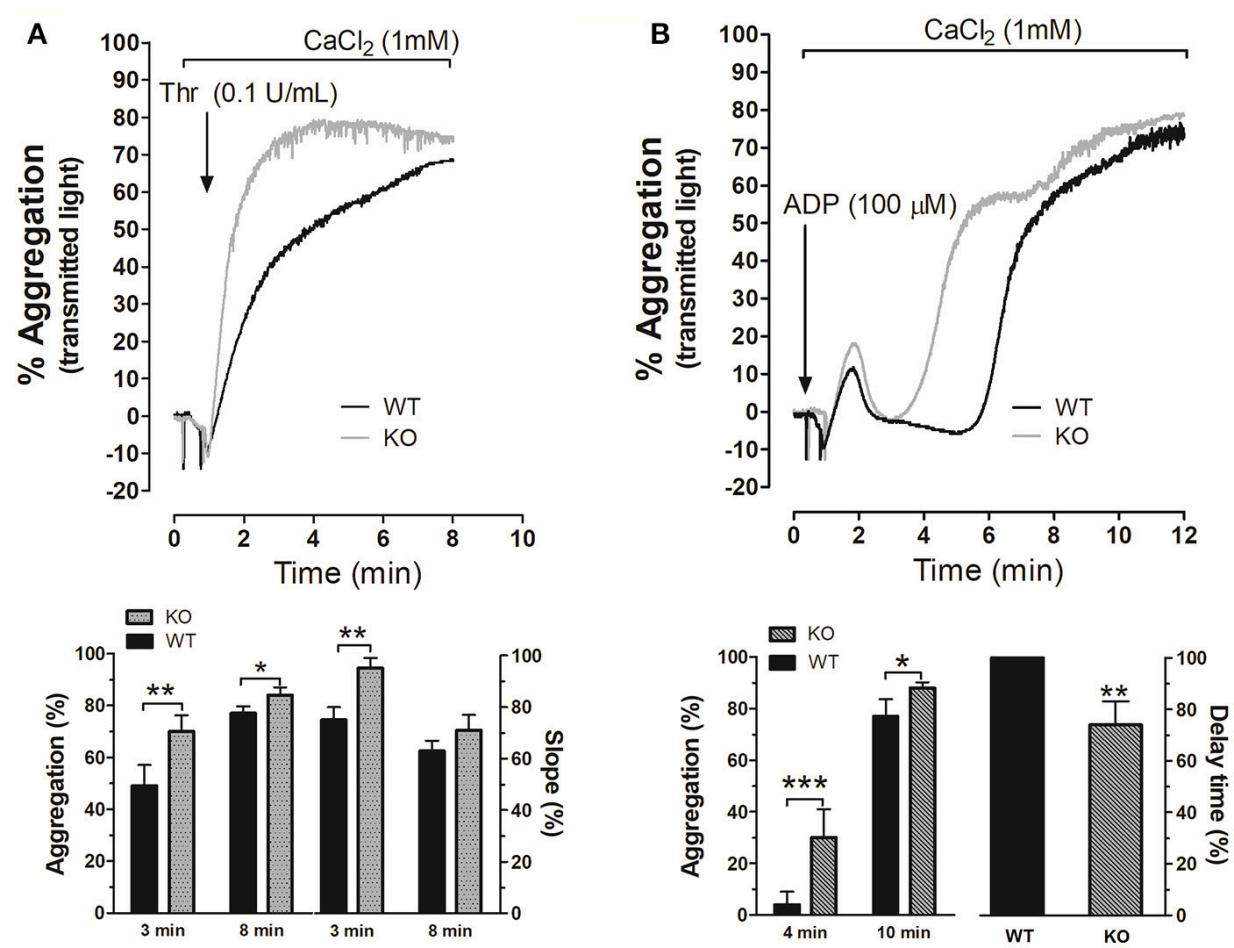
STC2 regulates platelet aggregation. Platelets drawn from WT (black traces) and STC2**-**deficient mice (gray traces) were isolated and suspended in Tyrode's buffer containing 1 mM Ca^2+^ and then stimulated with Thr **(A)** or ADP **(B)**. Aggregation of mice platelets was induced at a shear rate of 1,200 r.p.m. at 37°C in an aggregometer as described in the Materials and Methods section. Traces shown are representative of 14 separate experiments. Histograms represent the percentage of aggregation and slope for Thr-induced platelet aggregation or the percentage of aggregation and delay time for ADP-evoked response expressed as mean ± SEM. Delay time was presented as percentage of the value obtained in platelets from WT mice. *,**,***: represent *P* < 0.05, <0.01, and <0.001, respectively as compared to the corresponding WT value.

We next explored the possible role of STC2 in platelets aggregation. As depicted in Figure 2A, platelets from STC2-deficient mice exhibited enhanced rate and amplitude of aggregation in response to thrombin (Thr, 0.1 U/mL) compared to WT. The percentage of aggregation was 50% and 70% at 3 min, and 77% and 84% at 8 min in WT and STC2-deficient cells stimulated with Thr, respectively (*P* < 0,05); while the slope was 75 and 95% at 3 min and 63 and 71% at 8 min in platelets from WT and STC2-deficient mice, respectively. Similarly, platelets from STC2^−/−^ mice exhibited a significant difference in the percentage of aggregation 4 min after the addition of the weak agonist, ADP (100 μM; Figure 2B); while 10 min after the addition of ADP a slightly greater percentage of aggregation was still evident in STC2-defficient mice compared to WT mice. As depicted in the aggregation traces of Figure 2B and the below histogram (delay time), the initial phase of irreversible aggregation occurred significantly earlier in platelets from STC2-deficient mice, while the reversible aggregation almost occurred simultaneously in both strains. These findings indicate a certain degree of hyperactivity and hyperaggregability in platelets from STC2-deficient mice, which is consistent with the reduced bleeding time reported in Figure 1.

### Platelets from STC2^−/−^ mice presented altered Ca^2+^ homeostasis

**Figure 3:**
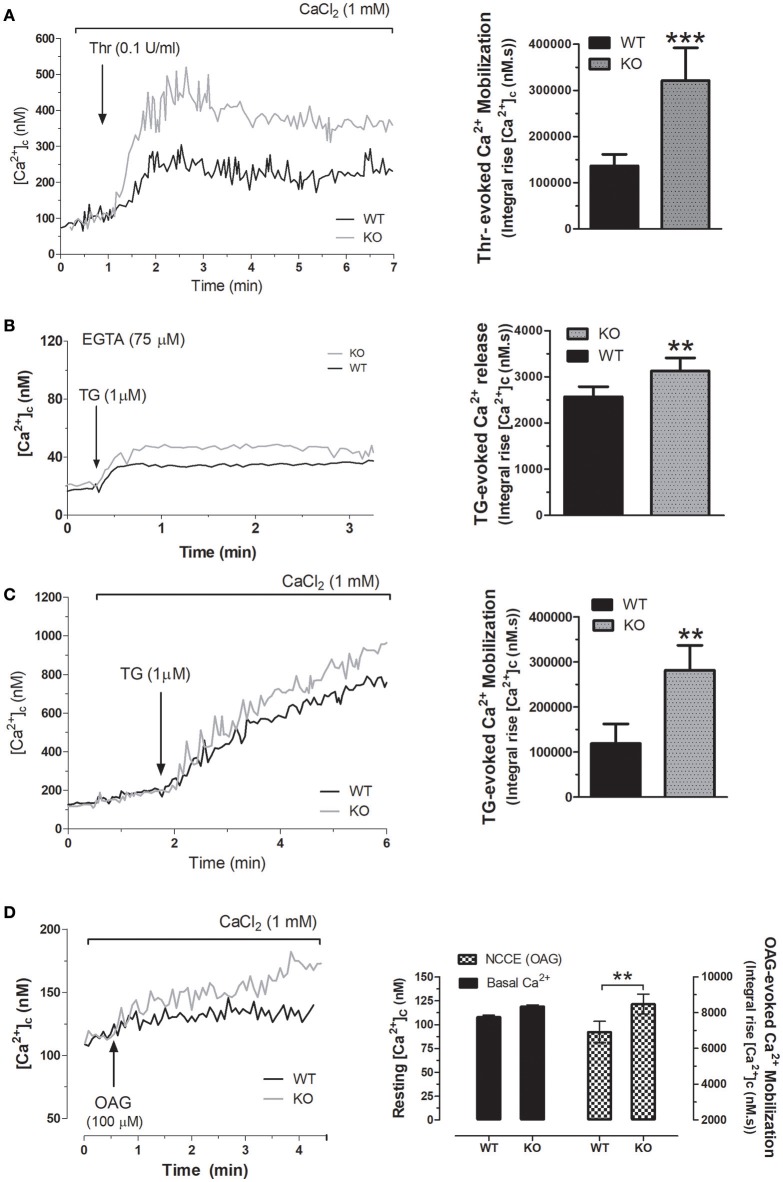
STC2 negatively regulates calcium entry. **(A)** Fura-2-loaded platelets from WT and STC2-deficient mice were suspended in a medium containing 1 mM Ca^2+^ (**A**, **C**, and **D**) or in a Ca^2+^-free medium (75 μM EGTA added; **B**). Cells were stimulated with Thr **(A)**, TG **(B,C)** or OAG **(D)**, as indicated. Calcium mobilization was determined as described in Material and Methods. Traces are representative of 8-10 independent experiments. Bar graphs represent agonist-evoked Ca^2+^ mobilization expressed as the mean ± SEM and presented as the integral of the rise in [Ca^2+^]_c_ above basal for 2½ min after the addition of the stimuli as described in Material and Methods. **, ***, *P* < 0.01, and 0.001, respectively, as compared to the response in platelets from WT mice.

As previously described by others, platelet function is regulated by several mechanisms, including intracellular Ca^2+^ mobilization. Hence, we have analyzed agonist-induced Ca^2+^ mobilization in platelets from WT and STC2^−/−^ mice. As shown in Figure 3A, in the presence of 1 mM extracellular CaCl_2_, the integral of Thr-evoked Ca^2+^ mobilization, estimated as described in Methods, in platelets from STC2-deficient mice was 308% greater than in platelets from WT mice (*P* < 0.001). Next, we have explored the mechanism underlying the exacerbated response to thrombin. First of all, we assessed Ca^2+^ release from the TG-sensitive stores using the SERCA inhibitor thapsigargin (TG), which induces passive Ca^2+^ efflux from the stores. The treatment of mice platelets with 1 μM TG in a Ca^2+^-free medium resulted in a sustained increase in [Ca^2+^]_c_ due to Ca^2+^ release from the internal stores. As shown in Figure 3B, TG-induced response was 27% greater in STC2-deficient cells. Inhibition of SERCA by TG reveals passive Ca^2+^ efflux from the ER, an event that is influenced by both the Ca^2+^ gradient across the ER membrane, which provides the driving force for Ca^2+^ efflux, and also the ER membrane permeability to Ca^2+^. As the response to TG, estimated as the integral of the rise in [Ca^2+^]_c_ over the resting level for 2½ min after the addition of TG, was found to be greater in STC2-deficient mice, thus suggesting that these cells have a greater ability to store Ca^2+^, we have further investigated the Ca^2+^ leakage rate in platelets from both strains. Our results indicate that slope of the rise in [Ca^2+^]_c_ upon stimulation with TG was similar in platelets from WT and STC2-deficient mice (1.05 ± 0.27 and 1.34 ± 0.18 nM/s in platelets from WT and STC2-deficient mice, respectively, *P* > 0.05; *n* = 8), thus suggesting that the leakage rate is similar in both strains.

Thr-evoked Ca^2+^-entry in human platelets mainly occurs via two different pathways, SOCE and non-capacitative Ca^2+^ influx (Jardin et al., 2009). Hence, we have further explored the mechanism involved in the exacerbated Thr mobilization in platelets from STC2-deficient mice. SOCE was assessed by depletion of the intracellular calcium stores using TG in the presence of extracellular calcium. Our results indicate that TG-evoked Ca^2+^ entry was significantly greater in STC2-deficient cells (Figure 3C); however, the differences in SOCE in platelets from both strains does not completely explain the magnitude of the different Ca^2+^ entry evoked by Thr. Therefore, our findings indicate that, in addition to the negative regulatory role of STC2 in SOCE, previously documented by others and confirmed by us in mice platelets, the exacerbated Thr-evoked Ca^2+^ mobilization might involve the enhancement of non-capacitative Ca^2+^ entry. To assess this possibility, platelets were stimulated with the diacylglycerol analog, OAG, a selective activator of non-capacitative Ca^2+^ entry channels (Hassock et al., 2002; Berna-Erro et al., 2012). As depicted in Figure 3D, treatment of platelets from WT mice with OAG (100 μM) in a medium containing 1 mM CaCl_2_ resulted in a sustained increase in [Ca^2+^]_c_. As expected, platelets from the STC2-deficient mice exhibited a greater increase in [Ca^2+^]_c_ in response to OAG; while we did not detect changes in the resting [Ca^2+^]_c_.

### STC2 modulates the interaction between STIM1 and orai1

**Figure 4:**
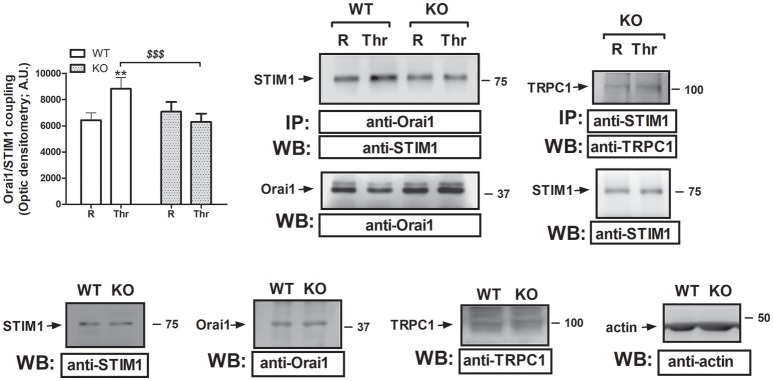
STC2 silencing impairs STIM1/Orai1 interaction. Murine platelets drawn from STC2-deficient (KO) and WT mice were either left untreated (R) or stimulated for 1 min with Thr (0.1 U/mL), and then lysed. Samples were then subjected to immunoprecipation using an anti-Orai1 or anti-STIM1 antibody, and subsequent Western blotting was performed using anti-STIM1 and anti-TRPC1 antibodies, as indicated. Membranes were reprobed with the immunoprecipitating antibody for protein loading control. *Bottom panels*, Platelet lysates from WT and STC2-deficient (KO) mice were subjected to Western blotting with anti-STIM1, anti-Orai1 or anti-TRPC1 antibody, as indicated. Membranes were reprobed with an anti-actin antibody as loading control. Blots are representative of 8-12 separate experiments. Bar graph represents Orai1-STIM1 coupling expressed as the mean ± SEM of the optical density in a.u. **P < 0.01 as compared to the response in resting platelets. ^$$$^*P* < 0.001 as compared to Thr-induced Orai1-STIM1 association in WT mice.

As mentioned above, SOCE requires physical interaction between the ER sensor STIM1 and the Ca^2+^ channel Orai1. Previous studies have revealed that STC2 regulates STIM1 expression and function, and subsequently, negatively modulates SOCE. Therefore, we have explored whether STC2 might be involved in the interaction between STIM1 and Orai1. As depicted in Figure 4, in platelets from STC2-deficient mice the interaction between STIM1 and Orai1 in response to Thr was significantly reduced. In addition, some authors have reported the existence of the interaction between STIM1 and TRPC1 in the absence of Orai1 in vascular smooth muscle cells. This mechanism was elicited by activation of PLCβ1, which requires changes in the [Ca^2+^]_c_ (Shi et al., 2017). Previously, we have also demonstrated interaction between STIM1 and TRPC1 in human platelets (Lopez et al., 2006). In platelets from STC2-deficient mice we have detected a 10% increase in the interaction between STIM1 and TRPC1 over the resting level after stimulation with Thr; therefore, a detectable interaction between STIM1 and TRPC1 takes place in platelets from STC2-deficient mice (see right hand side image of Figure 4). Furthermore, no differences were detected in the expression of the components of the SOCE mechanism in the platelet of STC2-deficient mice compared to those of WT. Therefore, Thr-evoked Ca^2+^ entry might involve STIM1-TRPC1 interaction in platelets from STC2-deficient mice.

### STC2 modulates the expression of orai3 in mouse platelets

**Figure 5:**
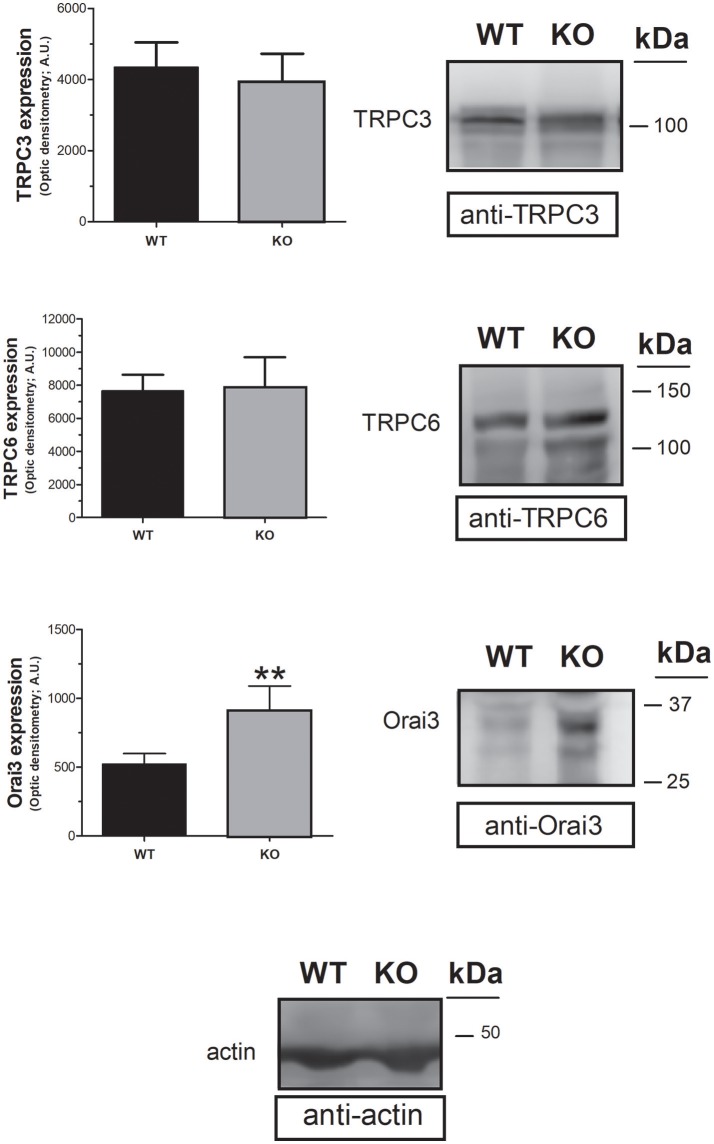
STC2 silencing upregulates Orai3 expression. Platelet lysates from WT and STC2-deficient (KO) mice were subjected to Western blotting with anti-TRPC3, anti-TRPC6 or anti-Orai3 antibody, and reprobed with anti-actin antibody, as indicated. Blots are representative of 6 separate experiments. Bar graph represents TRPC3, TRPC6 or Orai3 expression as the mean ± SEM of the optical density in a.u. **P < 0.01 as compared to the expression in platelets from WT mice.

In an attempt to assess the mechanism underlying the increase in non-capacitative Ca^2+^ entry in platelets from STC2-deficient mice, we have analyzed the expression of the non-capacitative channels at the protein level in the platelets of both strains. Previous studies have reported that TRPC3, TRPC6 and Orai3 are involved in non-capacitative entry (Berna-Erro et al., 2012; Harper et al., 2013). Hence, we have examined the expression of these channels in platelet lysates from WT and STC2-deficient mice by Western blotting using specific antibodies. As depicted in Figure 5, Western blotting of platelet lysates with anti-TRPC3 or anti-TRPC6 antibodies revealed that the expression of these proteins is not altered in the platelets of STC2-deficient mice with respect to WT. Interestingly, our results indicate that Orai3 expression is enhanced by 75% in platelets from STC2^−/−^ mice as detected by Western blotting with the anti-Orai3 antibody. This finding strongly suggests that STC2 modulates Orai3 expression in mice platelets. Furthermore, this observation might provide a mechanism for the enhanced non-capacitative Ca^2+^ influx in platelets from STC2-deficient mice.

## Discussion

Here we provide for the first time evidence supporting a role for STC2 in platelet aggregation and haemostasis. Using the murine model lacking STC2, we have found that these mice exhibited enhanced platelet response to strong and weak agonists, such as thrombin and ADP, respectively, and subsequently reduced bleeding time. Our results indicate that STC2 might modulate platelet aggregation through the regulation of Ca^2+^ homeostasis in these cells, as it was previously reported in other mammal cells (Zeiger et al., 2011). We have found that platelets from STC2-deficient mice exhibit a greater ability to store Ca^2+^ in the ER, although the leakage rate across the ER membrane is not significantly enhanced. Furthermore, our results indicate that STC2 modulate agonist-induced Ca^2+^ entry, so that the deficiency of STC2 results in exacerbated Ca^2+^ signals and, most likely, platelet hyperaggregability.

Thrombin evokes Ca^2+^ influx in platelets via SOCE and non-capacitative Ca^2+^ entry (Jardin et al., 2009). Our results indicate that STC2 plays a role supporting stimulated STIM1/Orai1 interaction, as Thr was unable to enhance STIM1/Orai1 association in platelets from STC2 KO mice. Although we have found a detectable increase in the STIM1-TRPC1 interaction upon stimulation with Thr, our findings indicate that it is unlikely that SOCE is enhanced, and, therefore, the enhanced Ca^2+^ influx induced by Thr in platelets from STC2-deficient mice is more likely mediated by enhanced non-capacitative Ca^2+^ entry. Our findings indicate that STC2 deficiency results in an impaired STIM1/Orai1 interaction, but also we have found that TG-induced Ca^2+^ entry in platelets from STC2 KO mice is significantly enhanced. This apparent discrepancy might be explained by the fact that TG has been found to activate non-capacitative Ca^2+^ entry in platelets through the release of autocrine factors (Harper et al., 2009); therefore, as for thrombin, the enhanced Ca^2+^ entry induced by TG in platelets from STC2-deficient mice might involve reduced SOCE and enhanced non-capacitative Ca^2+^ entry. In support of this hypothesis, we have found that non-capacitative Ca^2+^ entry evoked by OAG, a diacylglycerol analog, is enhanced in STC2-deficient mice, which provides evidence for a role of STC2 in the regulation of non-capacitative Ca^2+^ influx in these cells. In murine platelets, two main TRPC channels have been found to be activated in response to OAG, TRPC3 and TRPC6 (Harper et al., 2013). However, we have not detected significant changes in the protein expression of TRPC3 or TRPC6 channels in platelet from STC2-deficient mice. Orai3 has also been found to be involved in non-capactitative Ca^2+^ entry (Berna-Erro et al., 2012), and we have found that Orai3 expression is significantly enhanced in platelets from STC2-deficient mice. Although we have not directly addressed the involvement of Orai3 in Thr-induced non-capacitative Ca^2+^ entry, these findings suggest a possible mechanism for the enhanced activation of non-capacitative Ca^2+^ entry in these cells. The enhanced Orai3 expression in platelets from STC2-deficient mice is unlikely to be responsible for the enhanced Ca^2+^ release in response to TG, as a previous study has revealed that Orai3 is not involved in the TG-revealed ER Ca^2+^ leak (Leon-Aparicio et al., 2017).

We have recently suggested that STC2 might be involved in the pathophysiology of type 2 diabetes mellitus, where STC2 is downregulated (Lopez et al., 2017). We have previously reported that platelets from patients with type 2 diabetes mellitus exhibit both hyperactivity and hyperaggregability (Saavedra et al., 2004; Redondo et al., 2005; Lopez et al., 2013b). In the type 2 diabetic patients we found that non-capacitative Ca^2+^ entry is enhanced, while SOCE might be attenuated (Jardin et al., 2009, 2011). These findings resemble our current observations. Thus, our present results might provide an explanation to the platelet complications in diabetic patients as a reduction in STC2 expression might lead to platelet hyperaggregability due to enhanced Orai3 expression, although at present we cannot discard other possible mechanisms for the increase in non-capacitative Ca^2+^ entry in these cells. Summarizing, we provide for the first time evidence for a role of STC2 in platelet aggregation through the modulation of Ca^2+^ homeostasis.

## Author contributions

EL and LG-G performed most of the experiments with the same grade of responsibility. JP-G, CC, NB, and MG provided us with the mice blood samples, as well as they were in charge of the statistical analysis of data. GS performed a critical review of the manuscript, while JR and PR were in charge of conceptual and protocol design of the manuscript and wrote the manuscript.

### Conflict of interest statement

The authors declare that the research was conducted in the absence of any commercial or financial relationships that could be construed as a potential conflict of interest.
